# Biological Activity of Some Magnesium(II) Complexes of Quinolones

**DOI:** 10.1155/MBD.2000.101

**Published:** 2000

**Authors:** Iztok Turel, Andrej Šonc, Marija Zupančič, Kristina Sepčić, Tom Turk

**Affiliations:** 1 University of Ljubljana Faculty of Chemistry and-Chemical Technology Aškerčva 5 Ljubljana 1000 Slovenia; 2 University of Ljubljana Biotechnical Faculty Department of Biology Vena pot 111 Ljubljana 1000 Slovenia

## Abstract

A new magnesium complex of quinolone antibacterial agent was prepared. This new complex as well as a previously isolated complex of magnesium with ciprofloxacin were tested against various Gram positive and Gram negative microorganisms. Antimicrobial activities were evaluated using the agar diffusion test. The results have shown that all magnesium complexes are significantly less active than the parent quinolone drugs. It was also found that the activity of quinolones is reduced when the solutions of quinolones are titrated with magnesium ions.


### BIOLOGICAL ACTIVITY OF SOME MAGNESIUM(II) COMPLEXES OF QUINOLONES

Iztok Turel[1] *, Andrej Šonc[1], Marija Zupančič[1], Kristina Sepčić[2], and Tom Turk[2]

[1]University of Ljubljana, Faculty of Chemistry and Chemical Technology,
Aškerčeva 5, 1000 Ljubljana, Slovenia < iztok.turel@uni-lj.si>
[2]University of Ljubljana, Biotechnical Faculty, Department of Biology, Večna pot 111, 1000 Ljubljana, Slovenia

**Abstract**
A new magnesium complex of quinolone antibacterial agent was prepared. This new complex as well as a previously isolated complex of magnesium with ciprofloxacin were tested against various Gram positive and Gram negative microorganisms. Antimicrobial activities were evaluated using the agar diffusion test. The results have shown that all magnesium complexes are significantly less active than the parent quinolone drugs. It was also found that the activity of quinolones is reduced when the solutions of quinolones are titrated with magnesium ions.

**Introduction**
The quinolones represent a big family of synthetic antibacterial agents which are in broad use to cure several infectious diseases. Ciprofloxacin (cf=1-cyclopropyl-6-fluoro-4-oxo-7-(1-piperazinyl)-1,4-dihydroquinoline-3-carboxylic acid) is one of typical members of the group whereas the second quinolone used in this study (FCl=1-cyclopropyl-7-chloro-6-fluoro-4-oxo-1,4-dihydroquinoline-3-carboxylic acid) is only a precursor of cf and is not used in clinical practice (Scheme 1).

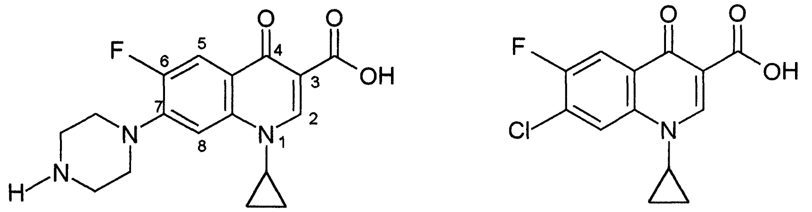

Scheme1: Chemical formulae of ciprofloxacin (left) and FCl (right).

It was found that the absorption of the quinolone drugs is lowered when they are consumed simultaneously with magnesium or aluminium antacids (Polk, 1989; Lomaestro and Bailie, 1991). Many other ions (Ca, Fe, Zn...) found in pharmaceuticals exert similar effects to quinolones (Shiba et al., 1994). The proposed reason for such behaviour could be the chelate bonding of the quinolone to the metal. This was one of the reasons that many authors started to study the interactions of metal ions and quinolones.

On the other hand, it seems that the role of metal ions is essential for the mode of action of these drugs. The activity of quinolones is due to the inhibition of the supercoiling of DNA catalyzed by the metalloenzyme DNA gyrase and it is also assumed that copper or iron complexes enable the bonding of the quinolone to the DNA (Shen and Pernet, 1985; Crumplin et al., 1980). Conflicting reports have appeared in the literature on the molecular details of drug-DNA and drug enzyme interactions. The first drug-DNA models have been proposed by Shen and co-authors (Shen et al., 1989) and have included hydrogen bond type interactions between the DNA unpaired bases and the quinolone, as well as a stacked dimerization of the drug. These models have been modified and imply a possible interaction between the C-7 substituent and the quinolone pocket on the B subunit of DNA gyrase (Morrissey et al., 1996). It was also discovered that metal ions, especially magnesium, are involved in such interactions (Palu et al., 1992). It was suggested that magnesium acts as a bridge between the quinolone and the phosphate groups of the DNA. Recently, another model based on the intercalation of quinolone into the double helix of DNA was proposed (Llorente et al., 1996). The structure is stabilized by the binding of the magnesium ion with oxygen atoms present in quinolone, a phosphate and a purine base of the DNA.

Our aim was to isolate new magnesium compounds of quinolones apart from those already prepared (Zupančič and Bukovec, 1996; Turel et al., 1996) and to compare the biological activity of these compounds against different microorganisms.

### Materials and Methods

*Synthesis*

$Mg(FCl^-)_2 H_2O$ (abbreviation MgFCl)

The quinolone (FCl) (154 mg) was dissolved in 35 mL of acetone. 70.1 mg of $Mg(NO_3)_2 6H_2O$ was added and the solution was stirred and slightly heated. After few minutes the white product precipitated. After few hours the product was filtered out and washed with water and acetone. Finally the product was left in a drier at 50 $^0$C for one hour. It was found that the isolated sample corresponds to formula $Mg(FCl^-)_2 H_2O$.

Found: C, 51.80; H, 2.91; N, 4.81; Mg, 4.15. $C_{26}H_{18}N_2O_7F_2Cl_2Mg$ requires C, 51.72; H, 2.98; N, 4.64; Mg, 4.03 %.

The same product was isolated if $MgCl_2{\cdot}6H_2O$ or $Mg(ClO_4)_2 xH_2O$ were used as reactants. All attempts to prepare the crystals suitable for X-ray analysis have failed.

The product is only sparingly soluble in water and in different organic solvents.

$[Mg(cf^-)_2(H_2O)_2]\ 2H_2O$ (abbreviation Mgcf)

The product Mgcf was prepared as reported elsewhere (Zupančič and Bukovec, 1996).

*Analyses and Physical Measurements*

The analyses of carbon, hydrogen and nitrogen were carried out on a Perkin-Elmer 204C microanalyzer.

The magnesium ion content has been determined by a titration with the ethylenediaminetetraacetic acid (EDTA). First the decomposition of the complex was performed as follows. 50.0 mg of sample MgFCl was dissolved in the mixture of 1.32 mL of concentrated nitric acid and 0.33 mL of sulphuric acid in a Kjehldahl flask. The mixture was heated till all the liquid evaporated. The residue was dissolved in the 100.0 mL of distilled water. The aliquot of the sample (25.0 mL) was transferred to the Erlenmeyer flask and the buffer solution (pH = 10) was added. The sample was titrated with 0.010 M EDTA solution and the indicator Eriochrome black T was used for the determination of the end point (Welcher, 1958).

Infrared spectra were recorded in the solid state on Nujol mulls between CsI windows on a Perkin-Elmer 1720X FT-IR spectrometer. Conditions: range 4000-220 $cm^{-1}$ resolution 2 $cm^{-1}$, number of scans 10.

*Antimicrobial activity tests*

The following bacterial strains were used: *Staphylococcus aureus, Staphylococcus epidermidis, Streptococcus salivarius, Streptococcus lactis, Streptococcus foecalis, Micrococcus luteus, Bacillus cereus, Bacillus subtilis, Aeromonas sp., Salmonella enteritidis, Escherichia coli, Enterobacter aerogenes, Proteus vulgaris, Klebsiella pneumoniae, Pseudomonas aeruginosa, Pseudomonas fluorescens* and *Shigella sonnei.* All the used bacterial strains were obtained from the local collection at the Department of Biology, University of Ljubljana.

Antimicrobial activities were evaluated using the agar diffusion test. The tested bacteria were allowed to grow overnight and their concentration was then determined. Bacterial culture was incorporated to Lauria Broth nutrient agar which was previously cooled to 42 °C. The final concentration of bacteria was approximately $5\times10^5$ CFU/mL (CFU – colony forming unit). Twenty millilitres of inoculated medium was poured into petri dishes and kept at 4 °C until use. Circles of agar ( $\Phi$ = 1 cm) were cut out from the cooled medium.

The MIC (minimal inhibitory concentration) values of Mgcf, FCl and MgFCl were determined, using ciprofloxacin and ciprofloxacin hydrochloride (cfHCl) as a reference substances. MIC represents the lowest concentration of an antibiotic that will inhibit the growth of a tested organism. For estimating MIC, the antibacterial substances were diluted gradually in 10 mM potassium phosphate buffer pH 7.4, containing 2 % DMSO. Hundred millilitres of each dilution were poured into the holes cut in the inoculated medium, after that the system was kept at 37 °C for 24 h. Finally, the diameters of inhibition zones were measured.

Additionally we wanted to check if there was any difference in the biological activity between the magnesium complexes and the samples in which we added free magnesium ions to the solutions containing ciprofloxacin.

The effect of magnesium ions on the antibacterial activity of ciprofloxacin was tested as follows: ciprofloxacin was diluted in 10 mM potassium phosphate buffer pH 7.4, containing 2 % DMSO. The final concentration of cf was 30 μg/mL. Fifty microlitres of ciprofloxacin were mixed with the same volume of different $MgCl_2$ dilutions in the same buffer. Agar diffusion test was used to determine the antibacterial activity, as described above. The tested microorganism was *S. aureus*.

### Results and Discussion

*The proposed structure of the complexes*

The infrared spectra of quinolones are quite complex and we only compared the most indicative vibrations of the samples used also in our previous studies (Turel et al., 1994; Turel et al., 1997; Turel et al., 1999). In the infrared spectrum of the free quinolone FCl we can assign the valence vibration of the carboxylic group $\nu(C{=}O)_c$ group at 1723 $cm^{-1}$ and the valence vibration of the ring carbonyl group at position 4 $\nu(C{=}O)_p$ at 1607 $cm^{-1}$. In the magnesium complex MgFCl a strong broad band appeared at 1626 $cm^{-1}$. The absence of the $\nu(C{=}O)_c$ and the shift of $\nu(C{=}O)_p$ vibrations could be the evidence that these groups are involved in the bonding to the metal. The changes in the IR spectra of Mgcf are similar and were described before (Zupančič and Bukovec, 1996). A similar infrared spectrum was found for the copper(II) complexes of ciprofloxacin

(Turel et al., 1994; Turel et al., 1999). The X-ray structure of this copper complex- $[Cu(cf)_2]Cl_2{\cdot}6H_2O$ revealed that copper is bidentately bonded to the quinolone through oxygen atom of the carboxylic group and oxygen atom of the ring carbonyl group. Such bonding was found also in some other metal complexes of quinolones (Baenziger et al., 1986; Chulvi et al., 1991; Ruiz et al., 1994; Koppenhoefer et al., 1995; Ruiz et al., 1995; Ruiz et al., 1997; Ruiz et al., 1998; Turel et al., 1999). According to these facts we assume that the bonding in magnesium complex of MgFCl is similar (Scheme 2).

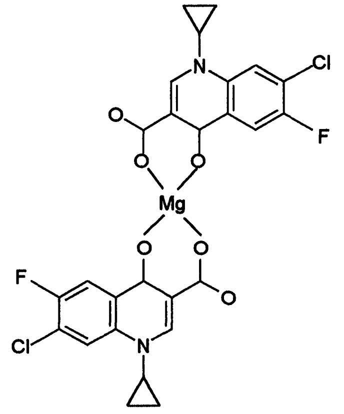

Scheme 2: The proposed bonding of magnesium to the quinolone.

**Table 1.** Antibacterial activity of cf, cfHCl, Mgcf, FCl and MgFCl. The activity is expressed as the MIC (minimal inhibitory concentration, which is the lowest concentration of an antibiotic that inhibits the growth of a tested organism). **MIC (μg/mL)**

| Microorganism | cf | cfHCl | Mgcf | FCl | MgFCl |
|---|---|---|---|---|---|
| *Staphylococcus aureus* | 1 | 1.2 | 1.2 | 2 | 30 |
| *Staphylococcus epidermidis* | 2.5 | 2.5 | 5 | 25 | 500 |
| *Streptococcus salivarius* | 0.02 | 0.02 | 0.2 | 1 | 7 |
| *Streptococcus lactis* | 3 | 3 | 6 | 60 | 70 |
| *Streptococcus foecalis* | 1 | 1.5 | 5 | 80 | 100 |
| *Micrococcus luteus* | 10 | 10 | 25 | 100 | 500 |
| *Bacillus cereus* | 1 | 1 | 1.5 | 0.6 | 3.5 |
| *Bacillus subtilis* | 0.5 | 0.5 | 0.5 | 0.5 | 1 |
| *Enterobacter aerogenes* | 1 | 1.5 | 2 | 5 | 50 |
| *Escherichia coli* | 0.05 | 0.05 | 15 | 2 | 25 |
| *Proteus vulgaris* | 0.1 | 0.1 | 0.25 | 0.75 | 5 |
| *Klebsiella pneumoniae* | 0.1 | 0.1 | 0.25 | 2 | 60 |
| *Pseudomonas aeruginosa* | 0.5 | 0.5 | 0.5 | 100 | 500 |
| *Pseudomonas fluorescens* | 0.25 | 0.25 | 1 | 25 | 500 |
| *Aeromonas sp.* | 0.025 | 0.025 | 0.4 | 0.05 | 0.4 |
| *Shigella sonnei* | 0.1 | 0.1 | 0.25 | 0.75 | 10 |
| *Salmonella enteritidis* | 0.25 | 0.25 | 0.25 | 1 | 10 |

*Antimicrobial activity tests*

The susceptibility of the bacteria to the tested agents is presented in Table 1. All the tested compounds showed approximately the same antibacterial activities against Gram positive and Gram negative bacteria. However, there is a difference in the susceptibility to the same antibiotic compound among different bacterial strains. The activity of Mgcf is about two folds lower than the activity of the reference compounds, cf and cfHCl.

The intermediate FCl and its magnesium complex MgFCl have remarkably lower antimicrobial activities comparing to the references. This is especially notable with MgFCl, which activity in some cases (*K. pneumoniae*) is even 30 fold lower as compared to FCl, or 600 fold lower as compared to cf. It seems that the introduction of magnesium into the ciprofloxacin molecule affects its antibacterial activity by diminishing it.

The same effect is observed when cf is titrated with increasing concentration of $MgCl_2$ (0-500 mM) (Figure 1). Magnesium decreases the antibacterial activity of ciprofloxacin even at very low concentrations. The loss of activity is especially notable at the $MgCl_2$ concentrations above 1 mM.

We can thus conclude that the activity of quinolones is remarkably lowered in the presence of magnesium ion both in the complexed or free form.

**Figure 1.** The effect of $Mg^{2+}$ on the antibacterial activity of ciprofloxacin. The antibiotic was titrated with increasing concentration of $MgCl_2$, and the activity was measured using agar diffusion test. The final concentration of ciprofloxacin was 15 μg/mL, and the tested microorganism was *Staphylococcus aureus.*

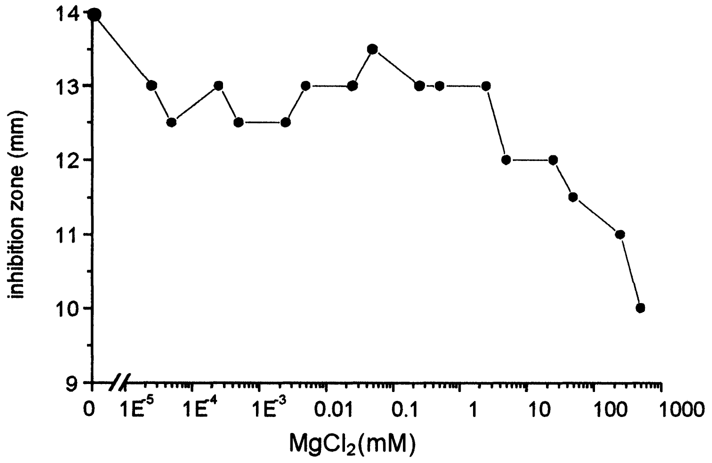

**Acknowledgement**

The Ministry of Sciences and Technology, Republic of Slovenia is thanked for financial support (COST-D8).

**References:**

1. Baenziger, N. C., Fox, C. L., Modak, S. L. *Acta Cryst.*, 1986; **C 42**: 1505.
2. Chulvi, C., Muñoz, M. C., Perell6, L., Ortiz, R., Arriortua, M. I., Via, J., Urtiaga, K., Amig6, J. M., Ochando, L.E. *J. Inorg. Biochem.*, 1991; **42**: 133.
3. Crumplin, G. C., Midgley J. M., Smith, J. T. *Top. Antibiot. Chem.*, 1980; **8**: 9.
4. Koppenhoefer, A., Hartmann, U., Vahrenkamp, H. *Chem. Ber.*, 1995; **128**: 779.
5. Llorente, B., Leclerc, F., Cadergen, R. *Bioorg. Med. Chem.*, 1996; **4**: 61.
6. Lomaestro, B. M., Bailie, G. R. *Ann. Pharmacother.*, 1991; **25**: 1249.
7. Morrissey, I., Hoshino, K., Sato, K., Yoshida, A., Hayakawa, I., Bures, M. G., Shen, L. L. *Antimicrob. Agents Chemother.*, 1996; **40**: 1775.
8. Palu, G., Valisena, S., Ciarrochi, G., Gatto, B., Palumbo, M. *Proc. Natl. Acad. Sci. U.S.A.*, 1992; **89**: 9671.
9. Polk, R. E. *Am.J. Med.*, 1989; **87**: 76(S).
10. Ruiz, M., Ortiz, R., Perell6, L., Garcia-Granda, S., Diaz, M.R. *Inorg. Chim. Acta*, 1994; **217**: 149.
11. Ruiz, M., Perell6, L., Ortiz, R., Castiñeiras, A., Maichle-Mössmer, C., Cantón, E. *J. Inorg. Biochem.*, 1995; **59**: 801.
12. Ruiz, M., Ortiz, R., Perell6, L., Latorre, J., Server-Carri6, J. *J. Inorg. Biochem.*, 1997; **65**: 87.
13. Ruiz, M., Perell6, L., Server-Carri6, J., Ortiz, R., Garcia-Granda, S., Diaz, M. R., Cantón, E. *J.Inorg. Biochem.*, 1998; **69**: 231.
14. Shen, L. L., Baranowski , J., Pernet, A. G. *Biochemistry*, 1989; **28**: 3879.
15. Shen L. L., Pernet, A. G. *Proc. Natl. Acad. Sci. U.S.A.*, 1985; **82**: 307.
16. Shiba, K., Sakamoto, M., Nakazawa Y., Sakai, O., *5th International Symposium on New Quinolones (Programme and Abstracts)*, 1994: 95.
17. Turel, I., Leban, I., Bukovec, N. *J. Inorg. Biochem.*, 1994; **56**: 273.
18. Turel, I., Leban, I., Zupančič, M., Bukovec, P., Gruber, K. *Acta Cryst.*, 1996; **C52**: 2443.
19. Turel, I., Bukovec, P., Quirós, M. *Int. J. Pharm.*, 1997; **152**: 59.
20. Turel, I., Golič, L., Ruiz Ramirez, O. L. *Acta Chim. Slov.*, 1999; **46**: 203.
21. Welcher, F. J., *The Analytical Uses of Ethylenediaminetetraacetic Acid*, D. van Nostrand Company, Inc., Princeton, 1958.
22. Zupančič M., Bukovec P. *Acta Pharm.*, 1996; **46**: 221.

